# Quantitative Relaxometry Metrics for Brain Metastases Compared to Normal Tissues: A Pilot MR Fingerprinting Study

**DOI:** 10.3390/cancers14225606

**Published:** 2022-11-15

**Authors:** Amaresha Shridhar Konar, Akash Deelip Shah, Ramesh Paudyal, Maggie Fung, Suchandrima Banerjee, Abhay Dave, Vaios Hatzoglou, Amita Shukla-Dave

**Affiliations:** 1 Department of Medical Physics, Memorial Sloan Kettering Cancer Center, New York City, NY 10065, USA; 2 Department of Radiology, Memorial Sloan Kettering Cancer Center, New York City, NY 10065, USA; 3 GE Healthcare, New York City, NY 10065, USA; 4 GE Healthcare, Menlo Park, CA 94025, USA; 5 Harlem Campus, Touro College of Osteopathic Medicine, New York City, NY 10027, USA

**Keywords:** MR Fingerprinting (MRF), relaxometry, quantitative MRI, normal-appearing brain tissue, brain metastases (BM)

## Abstract

**Simple Summary:**

Brain metastases (BM) are the most common central nervous system tumor in adults. Conventional qualitative magnetic resonance imaging (MRI), including T1- and T2-weighted imaging, assists in the diagnosis, treatment selection, monitoring, and prognostic determination of BM. Quantitative relaxometry (T1 and T2) measurements, besides other quantitative methods like diffusion- and perfusion-weighted MRI, are important for characterization of BMs, as well as elucidation of their underlying tumor tissue patho-physiology. New, rapid relaxometry methods, such as MR Fingerprinting (MRF), can provide tumor patho-physiology information within a single MRI acquisition and a clinically feasible timeframe. The purpose of the present pilot study was to estimate T1 and T2 metric values derived simultaneously from a new, rapid MRF technique and assess their ability to characterize BM and normal-appearing brain tissues. Our results showed that T1 (index for free water content) and T2 (index for tissue morphology) mapping may quantitatively characterize BMs. Our initial findings need to be validated in a larger patient cohort.

**Abstract:**

The purpose of the present pilot study was to estimate T1 and T2 metric values derived simultaneously from a new, rapid Magnetic Resonance Fingerprinting (MRF) technique, as well as to assess their ability to characterize—brain metastases (BM) and normal-appearing brain tissues. Fourteen patients with BM underwent MRI, including prototype MRF, on a 3T scanner. In total, 108 measurements were analyzed: 42 from solid parts of BM’s (21 each on T1 and T2 maps) and 66 from normal-appearing brain tissue (11 ROIs each on T1 and T2 maps for gray matter [GM], white matter [WM], and cerebrospinal fluid [CSF]). The BM’s mean T1 and T2 values differed significantly from normal-appearing WM (*p* < 0.05). The mean T1 values from normal-appearing GM, WM, and CSF regions were 1205 ms, 840 ms, and 4233 ms, respectively. The mean T2 values were 108 ms, 78 ms, and 442 ms, respectively. The mean T1 and T2 values for untreated BM (n = 4) were 2035 ms and 168 ms, respectively. For treated BM (n = 17) the T1 and T2 values were 2163 ms and 141 ms, respectively. MRF technique appears to be a promising and rapid quantitative method for the characterization of free water content and tumor morphology in BMs.

## 1. Introduction

Brain metastases (BM) are the most common central nervous system tumor in the United States, with an incidence of 9–17% in patients with cancer as well as high morbidity, mortality, and treatment costs [1]. Symptoms can include headaches, focal neurologic deficits, cognitive dysfunction, and seizures [2]. The most common primary cancers, accounting for more than four-fifths of BM, are lung (approximately 50%), breast (15–20%), melanoma (5–10%), renal (5–10%), and colon (1–6%) [2,3]. Overall survival rates are poor and differ significantly by primary diagnosis [4]. In recent decades, therapeutic innovations have prolonged survival for certain subsets of patients diagnosed with this previously lethal disease. Current treatment options include whole brain radiation therapy (WBRT), focal RT, stereotactic radiosurgery (SRS), surgery, chemotherapy, and newer hormone and molecular targeted therapies [5,6]. Imaging studies are critical for BM diagnosis, including computed tomography (CT) and magnetic resonance imaging (MRI).

Conventional MRI, including high-resolution multi-planar T1- and T2-weighted images, plays a vital role in BM diagnosis, treatment selection, monitoring, and prognosis determination [7]. However, this qualitative image analysis does not depict physiological changes and its utility is limited to the evaluation of tumor morphology [8]. Unfortunately, post-treatment changes in tumor volume and morphology do not necessarily correlate with patient outcomes [9]. A long-standing goal in the MRI community has been quantitative imaging, wherein properties of interest are quantitatively mapped, and image interpretation is both anatomical and numerical [10,11]. As treatment pathways evolve, there is a need for advanced quantitative imaging biomarkers (QIBs) that better guide treatment decisions by characterizing each BM’s structural, metabolic, and functional status [12]. Diffusion and perfusion are advanced MRI techniques that allow for quantification of tumor water diffusivity and vascular permeability [13,14,15]. While MR spectroscopy interrogates the molecular composition (e.g., 2-hydroxyglutarate) of tumors and has shown promise in noninvasively determining genetic subtypes [16]. Herein, the quantitative measurement of interest is relaxometry metrics, which also assess changes in tissue pathophysiology [17]. T1 and T2 mapping, for example, are indexes that determine free water content [18] and tissue morphology [19], respectively. Though they have shown potential for the determination of early tumor progression in the brain [20], long MRI acquisition times and limits of one acquisition per quantitative map [21,22,23] have prevented the day-to-day adoption of conventional quantitative relaxometry in clinical practice.

This challenge has led to the development of time-efficient quantitative MRI techniques that provide simultaneous multi-contrast images for relaxometry measurements in a single MRI acquisition sequence, thereby rapidly evaluating tissue characteristics in brain tumor patients in a clinically feasible time [24,25]. Ma et al. recently developed the MR fingerprinting (MRF) method, which uses the highly under-sampled pseudo-random acquisition method with dictionary matching to provide quantitative relaxometry measurements within a single sequence [26]. Vigorous testing of the MRF sequence has been carried out using the International Society for Magnetic Resonance in Medicine/National Institute of Standards and Technology (ISMRM/NIST) system phantom [27,28,29]. Human volunteer studies have also been performed to map healthy brain tissue on three major MRI vendor platforms, thereby measuring the bias and inherent reliability of T1 and T2 imaging metrics [26,30,31,32,33,34,35]. Badve et al. exhibited the ability of MRF–derived T1 and T2 metrics to differentiate between low-grade gliomas, high-grade gliomas, and brain metastases using first-order statistics from both solid tumor and surrounding peritumoral white-matter (WM) components [36]. In a subsequent study using the same data, Dastmalchian et al. found that radiomics with image feature analysis significantly improved differentiation between various tumor types [37]. In a separate study, Ma et al. demonstrated the utility of MRF beyond brain tumors, showing that relaxometry metrics could identify all 15 epileptogenic lesions compared to conventional MRI’s identification of 11 of 15 [38]. The purpose of this pilot study was to estimate T1 and T2 metric values derived simultaneously from newer, rapid MRF technique, as well as to assess their ability to characterize BM and normal-appearing brain tissues.

## 2. Materials and Methods

### 2.1. Phantom

A standard MRI system phantom procured from CaliberMRI (Boulder, CO, USA). It was co-developed by the International Society for Magnetic Resonance in Medicine (ISMRM) and the National Institute of Standards and Technology (NIST) to facilitate the conduct of qMRI studies, including and not limited to T1 and T2 relaxation times [39]. This phantom contains SI-traceable components and has been tested for stability and accuracy of its T1 and T2 values [40]. MRI system phantom consists of 14 vials in each T1 and T2 array, which are chemically formulated to provide wide range of T1 and T2 values, including the range of T1 and T2 values often found in gray and white matter of the brain. Vendor provided (VP) the Nuclear Magnetic Resonance (NMR) based reference T1 and T2 relaxation times for each vial.

### 2.2. Patient Cohort

The institutional review board approved this Health Insurance Portability and Accountability Act (HIPAA)-compliant prospective study for patients. Written informed consent was obtained from all eligible 27 patients with brain tumors, including 14 patients with evaluable BM lesions on MRI. There were a total of 21 BM lesions. The median age of the 14 BM patients in this pilot study was 53 years-old (age range, 25–72 years; 6 males and 8 females). All patients were enrolled between March 2019 and August 2021. The inclusion criteria were age ≥18 years and clinical or radiological diagnosis of BM. Exclusion criterion was any contraindication to MRI, such as a noncompatible cardiac pacemaker. Table 1 illustrates patient characteristics. Patients with BM underwent conventional clinical MRI and prototype MRF research sequence irrespective of their treatment group (untreated [n = 4] or treated [n = 10]). This study focused on MRF testing of BM patients. They were not scanned longitudinally but at a single time point. The therapy regimens for ten BM patients that underwent treatment were as follows: SRS n = 5, focal RT n = 2, WBRT n = 3. For these 10 treated patients the mean time interval from treatment to the MRI examination was 4 months, ranging between 2 to 9 months.

### 2.3. MRI Data Acquisition

ISMRM/NIST system phantom was scanned on GE (General Electric Healthcare, Waukesha, WI, USA) MRI system (Discovery 3.0 T MR750w) using an eight-channel brain array coil. The Gold Standard (GS) T1 measurements from the T1 arrays were acquired by the IR spin echo method with specific acquisition parameters as follows: inversion time (TI) = 50, 75, 100, 125, 150, 250, 500, 1000, 2000, 3000 ms; repetition time (TR) = 4500 ms; echo time (TE) = 7.34 ms; acquisition matrix 128 × 128; matrix reconstructed to 256 × 256; field of view (FOV) = 25 cm; slice thickness = 5 mm. The scan time for each TI measure-ment was approximately four minutes and the total scan time for GS T1 acquisition was around 40 min. The GS T2 measurements from the T2 array were obtained using a multi-ple single-echo spin echo method with the following acquisition parameters: TEs = 9, 12, 15, 20, 25, 30, 40, 45, 50, 60, 75, 80, 100, 120, 160 ms; TR = 5000 ms; acquisition matrix 128 × 128; matrix reconstructed to 256 × 256; FOV = 25 cm; slice thickness = 5 mm. The scan time of each TE measurement was approximately 21:30 (min:sec); the total scan time was approximately five hours. The nonlinear least-squares curve fitting was performed in MATLAB (The MathWorks. Inc., Natick, MA, USA). Due to the prolonged acquisition time of GS method and limited availability of the scanner, the repeated experiment was not performed. Whereas the phantom data was acquired using MRF over a period of 10 days in a coronal plane with an FOV of 25 cm. MRF parameters used for the phantom data acquisition were the same as the patient data acquisition detailed below.

All BM patients’ MRI examinations were performed on a 3T MRI scanner (Discovery MR750w, General Electric Healthcare, Waukesha, WI, USA) using a nineteen-channel head and neck unit (HNU) receive-only coil by GE Healthcare. In this study, we used conventional 2D axial T1-weighted (w) both pre- and post-contrast, as well as T2w fat-suppressed, fast spin-echo, and fluid attenuated inversion recovery (FLAIR) T2w images with a slice thickness of 3 mm and FOV of 20–24 cm. The acquisition parameters were as follows: T1w imaging with Repetition Time (TR) = 2000 ms, Inversion Time (TI) = 1101 ms, Echo Time (TE) = 25 ms, number of averages (NA) = 1, acquisition matrix size 320 × 224, reconstructed matrix size 320 × 256, and scan time of ~2.43 min; T2w imaging with fat-suppressed fast spin-echo using TR = 4796 ms, TE = 121 ms, NA = 1, acquisition matrix size 256 × 256, reconstructed matrix size 320 × 256, and scan time of ~2.27 min; FLAIR imaging with TR = 9946 ms, TE = 127 ms, TI = 2375 ms, NA = 1, acquisition matrix size 256 × 192, reconstructed matrix size 256 × 256, and scan time of ~5.13 min. In addition, pre- and post-contrast 3D images were acquired using brain volume (BRAVO) sequence for detection of all MRI visible lesions. It is an inversion recovery (IR)-prepared, fast spoiled gradient recalled echo (SPGR) sequence with parameters tuned to optimize brain tissue contrast with slice thickness = 1 mm. The additional parameters were TR = 6.6 ms, TE = 2.5 ms, TI = 450 ms, acquisition and reconstruction matrix size 256 × 256.

MRF data were acquired using a 2D steady-state free precession (SSFP) sequence with a variable density spiral (VDS) trajectory. A spiral trajectory (with 732 points) was rotated at a golden angle to achieve 89 interleaves. This process was repeated 11 times to acquire a total of 979 frames. The acquisition parameters were as follows: TR (minimum) = 14.7 ms and flip angle (FA) = ranging between 5° and 70°, as well as varied for 979 frames (per slice) [41]. Additional parameters included FOV = 25 cm, matrix size = 128 × 128, sampling bandwidth = ±250 kHz, TE = 2.2 ms, slice thickness = 5 mm, number of slices = 20, without gap and with a total scan time of 5.57 min.

Our image reconstruction pipeline includes a combination of compressed sensing with MRF which is more robust to low sampling ratio and is therefore more efficient in estimating MR parameters for all voxels of an object [42]. Highly under-sampled data acquired using the prototype MRF research sequence was reconstructed using a re-gridding algorithm followed by inverse Fourier transformation [43]. First, the acquired k-space samples projected onto a low-rank subspace determined by the singular value decomposition (SVD) of the MRF dictionary [44]. A fixed SVD value of 100 is used in the reconstruction of all the 14 patients’ datasets. The compressed k-space data transformed to the image space using a nonuniform fast Fourier transform, obtaining a set of subspace coefficient images for each receiver channel. [43]. The adaptive coil combination method uses multi-channel coil data, which combines them into a single image (per frame) [45]. These reconstructed images were then used for a voxel-wise pattern matching that determined the highest correlation between signal evolution and simulated dictionary [26]. The radiofrequency field (B1) non-uniformity was included in the dictionary.

The dictionary range and step size were matched with those reported by Jiang et al. [41]. Dictionary values (denoted as min: step: max) selected in this study were 20:10:3000 and 3000:200:5000 ms for T1, as well as 10:5:300 and 300:50:500 ms for T2.

### 2.4. MRI Tumor Regions of Interest Analysis

Regions of interest (ROIs) were delineated by a neuroradiologist with over seven years of experience in cancer imaging. All ROIs were drawn manually in a single plane and the conventional clinical qualitative MR images were used as cross-reference. To avoid partial volume effects, the BM lesions were delineated within the tumor boundaries, referring to additional and clinically acquired 1 mm slice thickness 3D MR images [46]. ROIs were drawn on T1 maps to encompass the solid enhancing portion of the tumor, excluding cystic and necrotic regions. We used the minimum size threshold for lesions ≥ 5 mm at our institution [47]. From this treated (n = 10) or untreated (n = 4) patient cohort, 21 BM lesions were delineated. The neuroradiologists reported a mean BM lesion size of 14 mm ranging from 5 mm to 26 mm. Therapy regimens varied for the ten patients who received treatment of their BM lesions: SRS n = 5, focal RT n = 2, WBRT n = 3. ROIs were also delineated in ipsilateral or contralateral normal-appearing GM (insular cortex), WM (frontal periventricular), and CSF (frontal horn) for eleven patients (4 untreated, 7 treated with SRS or focal RT). We did not assess the GM, WM, or CSF of the three patients who received WBRT as there can be microscopic changes in normal-appearing brain tissues after radiation exposure. MRF provides T1 and T2 maps simultaneously and 54 ROIs were manually drawn on either T1 or T2 maps. These 54 ROIs were drawn using the ImageJ software and saved as ROI files. These ROI files were then loaded on T1 and T2 maps separately to extract the relaxometry values cumulated to 108 measurements.

### 2.5. Statistical Analysis

We used Wilcoxon rank-sum test (WRST) to perform univariate analysis, comparing two independent samples to identify differences between the untreated and treated patients’ groups with an unequal number of samples (variables) for both the T1 and T2 values of BM measured. WRST is also called the Wilcoxon-Mann–Whitney test and Mann–Whitney U test. For this analysis, the significance level was set to *p* ≤ 0.05. T1 and T2 values were obtained from normal-appearing GM, WM, and CSF. The median, mean ± standard deviation (SD), and range of these metric values were also reported (Supplementary Table S1). Further, the T1 and T2 values of both untreated and treated groups were compared with the normal-appearing brain tissues.

## 3. Results

Table 2 reports the MRF estimated mean T1 and T2 values from phantom over a period of 10 days, as well as the relative percentage difference, between the MRF estimated and vendor provided (VP), MRF and gold standard (GS) values. The relative percentage difference between the above methods for T1 values showed a maximum of 9.4% between MRF and VP, and 5.7% between MRF and GS. Similarly, the percentage difference between these methods for T2 values showed a maximum of 34.4% between MRF and VP, and 47.4% between MRF and GS.

**Figure 1:**
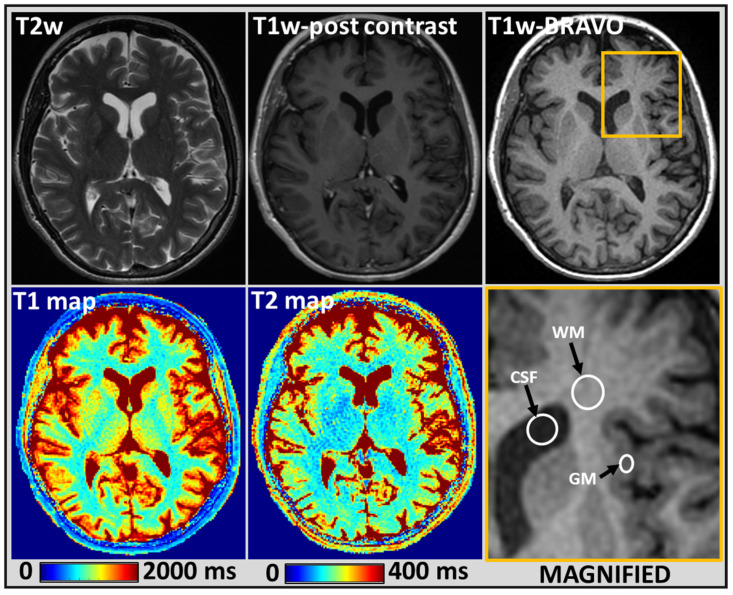
Representative MR images showing normal-appearing brain tissue from a 52-year-old female patient treated with focal RT and had a history of primary melanoma: Conventional T2-weighted (w), post-contrast T1w and BRAin VOlume (BRAVO) images depicting the anatomical structures (**top row**). T1 and T2 maps estimated using MRF for a single slice exhibiting gray matter (GM), white matter (WM), and cerebrospinal fluid (CSF) (**bottom row**). The yellow outlined region on a BRAVO image was magnified, and the placement of region of interest (ROI) is in white for normal-appearing GM, WM, and CSF. The MRF estimated mean T1 values for these normal-appearing GM, WM, and CSF regions were 1204 ms, 842 ms, and 4190 ms, respectively, and the mean T2 values were 108 ms, 78 ms, and 442 ms.

The final analysis was performed on 108 measurements obtained from the MRF estimated T1 and T2 maps. Figure 1 shows ROI placement on MRF estimated T1 and T2 maps for the normal-appearing GM, WM and CSF on a reference BRAVO image of a representative patient.

**Figure 2:**
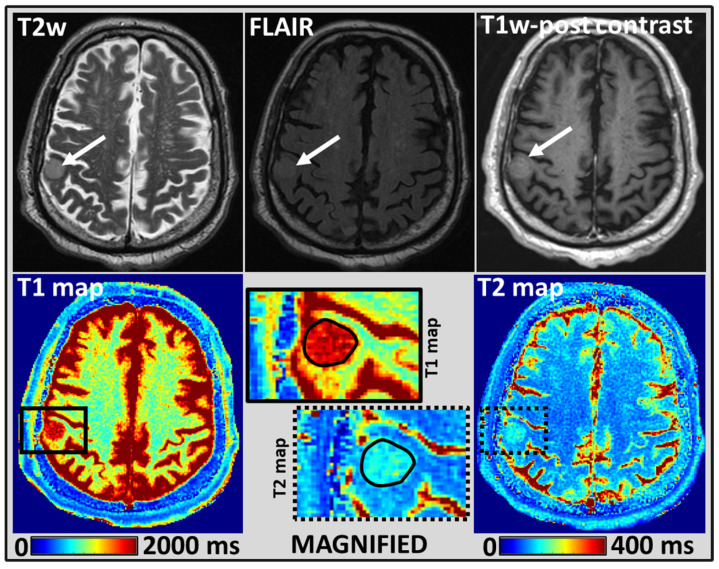
Representative MR images from a 72-year-old male patient with BM in the right parietal lobe treated with SRS and had a history of primary lung cancer: T2w (arrow pointing to the lesion), T2w fluid-attenuated inversion recovery (FLAIR), and T1w post-contrast images showing BM lesion at right parietal lobe (**top row**). MRF-derived quantitative T1 and T2 maps (**bottom row**). A black solid and dotted rectangle region on T1 and T2 maps, respectively, were magnified, and the placement of a manually drawn ROI for the BM lesion was shown in a solid black line. The MRF-derived T1 and T2 values at the BM lesion were 2355 ms and 154 ms, respectively.

**Figure 3:**
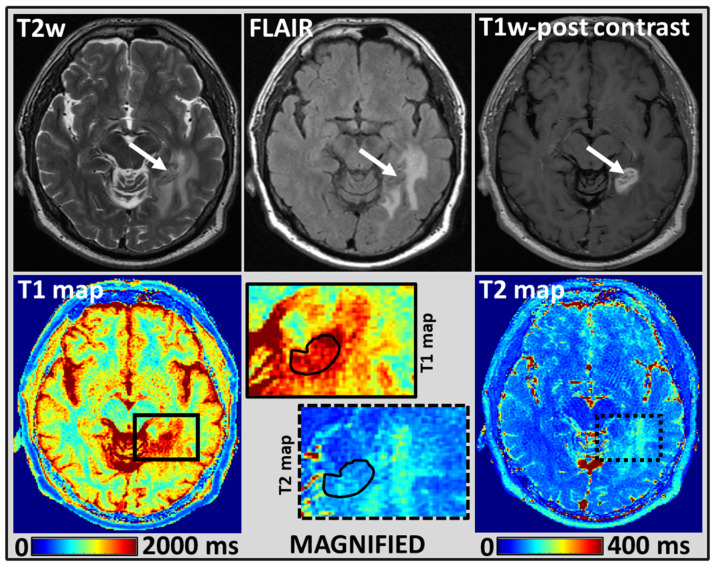
Representative images from a 59-year-old female patient treated with focal RT and had a history of primary lung cancer: T2w (arrow pointing to the lesion), T2w FLAIR, and T1w post-contrast images from the clinical scan, which demonstrate a left temporal lobe enhancing metastasis (**top row**). A black solid and dotted rectangle region on T1 and T2 maps, respectively, were magnified, and the placement of a manually drawn ROI for the BM lesion was shown in a solid black line. MRF-derived quantitative T1 and T2 maps (**bottom row**). The T1 and T2 values from the treated BM lesion were 1906 ms and 106 ms, respectively.

Similarly, Figure 2 and Figure 3 show the shape of ROI and placements on MRF estimated T1 and T2 maps for BM lesions from two different representative patients.

**Figure 4:**
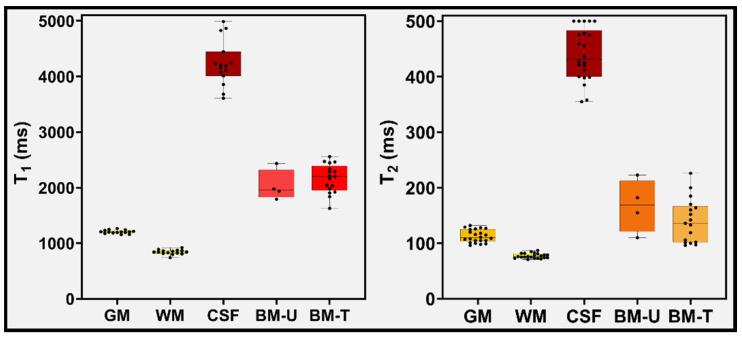
Analytics of the T1 and T2 values for normal-appearing GM, WM, CSF, untreated (U) and treated (T) BM using the T1 and T2 maps generated from MRF method. Boxes represent the interquartile range, whiskers represent the range of all values, and the horizontal line within the box is the median value.

For the normal-appearing tissues, the mean values for T1 and T2 of GM, WM and CSF were 1205 ms and 108 ms, 840 ms and 78 ms, and 4233 ms and 442 ms, respectively (see Figure 4 and Supplementary Table S1).

WRST performed for the mean T1 and T2 values estimated for the normal-appearing WM and BM lesions showed significant differences (*p* < 0.05); in both untreated (n = 4) and treated (n = 17) groups. For the BM lesions from the untreated group, the mean T1 and T2 values were 2035 ms and 168 ms, respectively. Similarly, the treated group’s mean T1 and T2 values were 2163 ms and 141 ms (see Figure 4 and more details in supplementary Table S1). However, the mean T1 and T2 values for BM lesions between the untreated and treated groups, showed no significant difference (*p* > 0.05).

**Figure 5:**
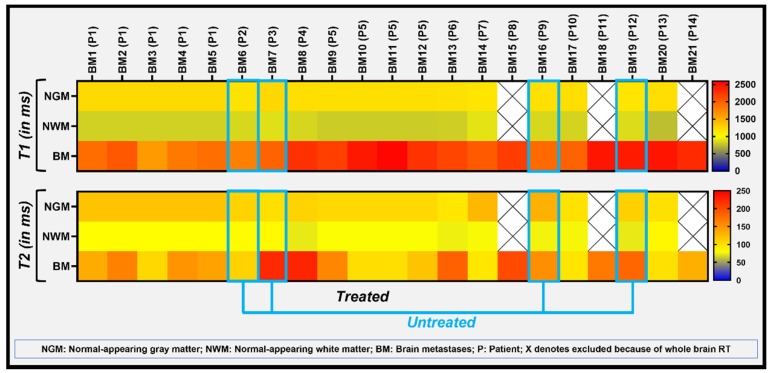
The heatmap exhibits the T1 and T2 metric values of all BM lesions as well as normal-appearing GM and WM. All 21 metastases from 14 patients were separately analyzed (patients were coded as “P” and metastases lesion as “BM”) and two patients (P1 and P5) had more than one metastasis (5 and 4 BM, respectively).

The heat map exhibits the heterogeneity in the measured T1 and T2 values between the 5 BM lesions in patient 1 (Figure 5). T1 values were more homogeneous between patients compared to T2 values, with exception of patient 1, exhibiting slightly lower T1 values (Figure 5). This patient had received SRS treatment and had a history of primary lung cancer. Similarly, 3 of the 4 untreated BMs showed slightly lower T1 values. As a note, the primary tumors for these patients were, colon cancer, melanoma, ovarian cancer, and lung cancer, respectively. The difference in T1 and T2 values between BMs and the normal-appearing GM and WM can be visualized and appreciated in the heatmap obtained from 86 ROI placements.

## 4. Discussion

Quantitative MRI-derived T1 and T2 metrics reflect local tissue properties, which provide valuable information for the assessment of tumor response to oncologic treatment [8,11,36]. T1 and T2 relaxation properties depend on macromolecule contents and the extent of water binding to these macromolecules [17,48]. Conventional methods of T1 and T2 measurement using MRI are time-consuming and challenging to apply in the clinical workflow. Recent developments in rapid, quantitative methods, such as the MRF-based technique have allowed for simultaneous measurement of T1 and T2, which have shown promise in the detection of lesions in patients with brain tumors [36,49], multiple sclerosis [50], and epilepsy [38]. No study has yet been reported comparing untreated and treated brain metastases (BM) and normal-appearing brain tissue using this new, rapid MRF technique. Our pilot MRF study was the first to propose such an investigation with analysis of 108 measurements (T1 and T2 combined) and reported significant difference between T1 and T2 values measured in BMs (untreated and treated) and normal-appearing white matter. The heatmap demonstrated tumor heterogeneity within the solid parts of multiple treated BMs in the same patient and between different patients (untreated and treated) using the MRF estimated T1 and T2 values. This study emphasized the importance of tissue relaxation time measurements for the understanding of tumor pathophysiologic changes in BMs, which may ultimately improve patient management after validation in a larger BM patient cohort.

Badve et al. reported that MRF-based T1 and T2 metric values showed significant differences between solid tumor regions of lower-grade gliomas (n = 6) and metastatic brain lesions (n = 8) [36]. In this study, T1 and T2 metrics values were 1324 ms and 105 ms, respectively, at pre-treatment in 8 patients with BM [36]. In the present study, mean T1 and T2 values of untreated BM lesions (n = 4) were T1 = 2035 ms and T2 = 168 ms. Our study’s higher T1 relaxation times could be associated with the differences in the primary tumor site for BMs. The difference in metric values may be attributed to the fact that experiments were performed on two different vendors’ MRI scanners with slight differences in acquisition parameters.

A previous study using conventional relaxometry techniques demonstrated differences between measured T1 and T2 values between the treated and untreated BM groups and this could be attributed to treatment related effects [51]. Lower T2 values in the treated BM group could be due to treatment-related changes to the microvasculature [52]. The leakage of blood in tissue may affect the T1 and T2 measurements, as shorter relaxation times have been associated with higher iron concentrations in the blood [53]. Treated BMs may increase in size over time despite absence of tumor growth, therefore conventional T1 and T2-weighted imaging cannot reliably differentiate between radiation necrosis and tumor [54,55]. Advanced imaging techniques including perfusion and diffusion MRI, and MR spectroscopy are often utilized for better characterization of lesions following treatment [13,14,15,16]. In some of our treated BMs cohort, on the T2-weihted and T1 post-contrast images, a radiation necrosis cannot be excluded. This might be the reason for differences or heterogeneities between relaxometry values measured. Badve et al. reported T1 and T2 values of 911 ms (T1 value) and 72 ms, respectively, for the normal-appearing contralateral WM [36]. In another study, Badve et al. showed slight variation in the measured T1 and T2 values measured at different location of white matter region, sex, and age of study population [56]. The mean T1 and T2 values obtained in this pilot MRF study for the WM were 840 ms and 78 ms, respectively in 11 BM patients.

Several T1 and T2 imaging techniques have been developed previously for quantification of MR relaxometry values. MRF-based T1 and T2 values were compared with literature values obtained with the established T1 and T2 mapping techniques for normal-appearing brain tissue. Gelman et al. used the TOne by Multiple ReadOut Pulses (TOMROP) sequence, a variant form of the Look-Locker method [22], to measure T1 relaxation values of brain tissue from twelve healthy adults on a 3T MRI scanner. The estimated mean T1 value of the frontal WM using TOMROP was 846 ms [22]. Utilizing a saturation recovery method with a variable TR spin-echo imaging, Wansapura et al. reported a mean T1 value of 838 ms for frontal WM in 19 healthy volunteers [57]. Using a gradient-echo sampling, Gelman et al. reported a T2 value of 55.8 ms for frontal WM from 6 healthy adults. Wansapura et al. reported a T2 value of 74 ms for frontal WM using the multiple spin-echo measurements [57]. Pirkl et al. utilized the 3D multiparametric quantitative transient-state imaging (3D QTI) method to measure T1 and T2 values in nine glioma patients [58]. 3D QTI T1 and T2 values were 903 ms and 46 ms, respectively, for WM. Previously reported T1 and T2 values for frontal GM were 1322 ms and 110 ms [57]. 3D QTI derived T1, and T2 values were 1353 and 66 ms, respectively, for GM [49]. In the present study, MRF-generated mean T1 and T2 values were 1205 ms and 108 ms for GM, and 840 ms and 78 ms for WM. Our MRF-estimated T1 and T2 values fall within the expected range for 3T MRI based on published values at 1.5 and 4 T [50,51,52]. Overall, the conventional methods provide single tissue contrast property which are relatively time-consuming. Thus, rapid MRF is a promising method for simultaneous quantification of multiple tissue parameters.

We recently reported the mean T1 and T2 values for BMs using a new, rapid, synthetic MRI (MAGnetic resonance imaging Compilation (MAGiC)) method [59]. The relaxometry values estimated with MAGiC were slightly lower on comparison to MRF results mentioned above. MAGiC and MRF are two prominent new sequences for rapid MR relaxometry and the former uses a multiple-dynamic multiple-echo (MDME) sequence for data acquisition and synthetic image reconstruction, including post-processing which is different from MRF as detailed in the method section [59,60]. The differences in the acquisition and reconstruction approaches could be attributed to the marginal variation observed in the mean T1 and T2 values. The relative percentage difference between the MRF and MAGiC estimated T1 and T2 values were 5% and 38% in GM, 20% and 22% in WM, and 1% and 13% in CSF. The mean T1 and T2 values estimated in the present study for the normal-appearing GM, WM, CSF regions and BM were compared with some of the previously reported values [24,26,30,32,36,59,61,62,63] (see Supplementary Table S2).

Several advanced MR imaging techniques have aimed to characterize the functional, physiological, and metabolic status of brain neoplasms, including MR perfusion, diffusion, and spectroscopy [64,65,66]. Results using the above techniques have been validated in multiple studies, demonstrating their ability to differentiate between various brain tumor subtypes, benign versus malignant disease, and provide insight into underlying tumor biology that can guide treatment [67]. However, these separate imaging acquisitions each have their own technical complexity. For example, MR perfusion involves the injection of a contrast agent and advanced modeling of the acquired data with a balance between spatial and temporal resolution [68]. MRF in comparison, is a rapid single acquisition sequence providing multi-contrast images without the use of a contrast injection. MRF is promising to become a clinically valuable tool, and the derived relaxometry related imaging biomarkers may aid in characterizing BMs [12]. Combining one or more of these advanced imaging techniques with conventional imaging can provide a wealth of clinically valuable information about BMs.

This pilot study had several limitations, including the heterogeneity of BMs from different primary sites, diverse treatment regimens, and a small sample size, particularly in the untreated group we had only four BM. We follow the institutional guidelines of small sample size for implementation and testing of a new MRI research prototype sequence (such as MRF) that is not yet part of clinical brain imaging. 3D MRF developments are underwayto overcome the limitation associated with 2-D MRF, such as partial volume effects. In our 2D MRF study, the slice thickness was 5 mm to get an optimal signal based on literature [36,59]. Further, imaging findings were not correlated with molecular or genomic markers as this was beyond the scope of our study. A larger BM patient cohort study is warranted to draw more definite conclusions and validate our initial results. Time interval between treatment and the MRI examination varies between the patients and this might have effect on estimated T1 and T2 values because of difficulty in differentiation between radiation necrosis and residual tumor. In addition, diffusion- and perfusion-MRI was performed in a conventional clinical exam, and it was not part of this pilot study.

## 5. Conclusions

In the present pilot study, MRF-based relaxometry T1 and T2 values distinguished BM from normal-appearing GM and WM. This rapid and simultaneous quantitative imaging technique may become a promising addition to conventional brain imaging for clinical oncological applications.

## Figures and Tables

**Table 1:** Patient characteristics.

Characteristics	Value
Total Patients	14
Total number of brain metastases lesions	21
Demographics Median age (Y) Age range (Y) Male/Female	53 25–72 6/8
Location of primary tumor	
Lung Colon Melanoma Other	6 2 3 3
Untreated/Treated	4/10

**Table 2:** Estimated T1 and T2 values from three different methods using MRI system phantom.

**Vial**	**T1 (ms)**			**Relative Difference (%)**		
	**VP**	**GS**	**MRF**		**MRF and VP**	**MRF and GS**
1	1838	1780	1881		2.3	5.7
2	1398	1351	1301		6.9	3.7
3	998.3	958	927		7.1	3.2
4	725.8	678	671		7.6	1
5	509	483	461		9.4	4.6
6	367	346	352		4.1	1.7
7	258.7	242	237		8.4	2.1
**Vial**	**T2 (ms)**			**Relative Difference (%)**		
	**VP**	**GS**	**MRF**		**MRF and VP**	**MRF and GS**
1	645.8	537	637		1.4	18.6
2	423.6	357	440		3.9	23.2
3	286	246	288		0.7	17.1
4	184.8	163	206		11.5	26.4
5	134.1	118	155		15.6	31.4
6	94.4	82	115		21.8	40.2
7	62.5	57	84		34.4	47.4

VP: Vendor Provided; GS: Gold Standard; MRF: Magnetic Resonance Fingerprinting.

## Data Availability

The data presented in this study will be provided upon reasonable request.

## Supplementary Materials

The following supporting information can be downloaded at: https://www.mdpi.com/article/10.3390/cancers14225606/s1, Table S1: T1 and T2 values from normal-appearing tissue and brain metastases, Table S2: Comparison of our MRF estimated T1 and T2 values from normal-appearing tissue and brain metastases with the published study.
