# Supplementary material for: Quantitative Relaxometry Metrics for Brain Metastases Compared to Normal Tissues: A Pilot MR Fingerprinting Study
Source: Cancers (Basel). 2022 Nov 15;14(22):5606. doi: 10.3390/cancers14225606 (PMC9688653; doi:10.3390/cancers14225606)
Supplement: Supplementary file 1 [file cancers-14-05606-s001.zip › cancers-1943831-supplementary.pdf]

**Supplementary Table S1:** T1 and T2 values from normal-appearing tissue and brain metastases

| Tissue | T1 (ms) | | | T2 (ms) | | |
|---|---|---|---|---|---|---|
| | **Mean ±SD** | **Median [min, max]** | **CI (95%) [Upper, Lower]** | **Mean ±SD** | **Median [min, max]** | **CI [Upper, Lower]** |
| **WM** | 840±42 | 842 [742, 919] | [864, 817] | 78±4 | 77 [73, 85] | [80, 75] |
| **GM** | 1205±31 | 1204 [1155, 1266] | [1223, 1188] | 108±11 | 107 [96, 132] | [114-103] |
| **CSF** | 4233±406 | 4190 [3610, 4990] | [4458, 4009] | 442±56 | 459 [355, 500] | [473, 411] |
| **BM-Untreated** | 2035 ±278 | 1957 [1792, 2435] | [2478, 1593] | 168 ±47 | 169 [110, 223] | [243, 92] |
| **BM-Treated** | 2163 ±258 | 2202 [1629, 2558] | [2296, 2030] | 141 ±40 | 136 [96, 226] | [161, 120] |

**Supplementary Table S2:** Comparison of our MRF estimated T1 and T2 values from normal-appearing tissue and brain metastases with the published study.

| **Tissue** | **Metric** | **Current Study** | **MAGiC [59]** | **MRF [26]** | **MRF [32]** | **MRF [30]** | **MRF [36]** | **Other [24, 61–63]** |
|---|---|---|---|---|---|---|---|---|
| **WM** | T1 (ms) | 840±42 | 701±39 | 685±33 | 954±15.4 | ~800-1000 | 911±39 | 608-756 |
| | T2 (ms) | 78±4 | 64±4 | 65±4 | 39±1 | ~50-70 | 72±6 | 56-81 |
| **GM** | T1 (ms) | 1205±31 | 1143±28 | 1180±104 | 1372±13 | ~1200-1350 | - | 998-1304 |
| | T2 (ms) | 108±11 | 78±2 | 97±6 | 53±1 | ~60-120 | - | 78-98 |
| **CSF** | T1 (ms) | 4233±406 | 4206±226 | 4880±379 | 2330±70 | ~1800-2200 | - | 4103-5400 |
| | T2 (ms) | 442±56 | 390±93 | 550±251 | 214±23 | ~-110-240 | - | 1800-2460 |
| **Untreated BM** | T1 (ms) | 2035±278 | 1868±298 | - | - | - | 1324±273 | - |
| | T2 (ms) | 167±47 | 100±17 | - | - | - | 105±27 | - |
| **Treated BM** | T1 (ms) | 2163±218 | 2211±269 | - | - | - | - | - |
| | T2 (ms) | 140±40 | 114±20 | - | - | - | - | - |
